# Suppressor of cytokine signalling-2 limits IGF1R-mediated regulation of epithelial–mesenchymal transition in lung adenocarcinoma

**DOI:** 10.1038/s41419-018-0457-5

**Published:** 2018-03-20

**Authors:** Yue Zhou, Zhilei Zhang, Ning Wang, Jizheng Chen, Xu Zhang, Min Guo, Li John Zhong, Qian Wang

**Affiliations:** 1 Department of Thoracic Surgery, First Affiliated Hospital of Nanjing Medical University, Nanjing, 210029 China; 2 Jiangsu Province Key Lab of Human Functional Genomics, Department of Biochemistry and Molecular Biology, Nanjing Medical University, Nanjing, 210029 China; 3 State Key Lab of Virology, Wuhan Institute of Virology, Chinese Academy of Sciences, Wuhan, 430071 China; 4 State Key Laboratory of Natural Medicines, School of Life Science and Technology, China Pharmaceutical University, Nanjing, Jiangsu 210009 China

## Abstract

Non-small cell lung cancer (NSCLC), including adenocarcinoma and squamous cell carcinoma, is the leading cause of death from lung malignancies and has a poor prognosis due to metastasis. Suppressor of cytokine signalling-2 (SOCS2), a feedback inhibitor of cytokine signalling, has been shown to be involved in growth control. Here, we show that SOCS2 were significantly downregulated in tumour foci in NSCLC patients. The expression levels of SOCS2 significantly correlated with clinical stage, lymph node metastasis, histological subtype and survival time. In particular, the decreased expression of SOCS2 significantly associated with advanced pathological stage, lymph node metastasis and shorter overall survival in lung adenocarcinoma patients. In vivo animal results showed that overexpressed SOCS2 attenuated the metastatic characteristics of lung adenocarcinoma, including by inhibiting the epithelial–mesenchymal transition (EMT). Further functional studies indicated that insulin-like growth factor 1 (IGF1)-driven migratory and invasive behaviours of lung adenocarcinoma cells can be partially suppressed by exogenous SOCS2 expression. Investigations into the mechanism of action revealed that SOCS2 inhibits EMT by inactivating signal transducer and activator of transcription 3 (STAT3) and STAT5 via the competitive binding of SOCS2 to the STAT binding sites on IGF1R. Altogether, our results reveal an important role for SOCS2 dysregulation in the pathogenicity of lung adenocarcinoma, suggest its potential use as a biomarker for diagnosing lung adenocarcinoma, and paves the way to develop novel therapy targets as the axis of SOCS2–IGF1R–STAT in lung adenocarcinoma.

## Introduction

Lung cancer, one of the most common malignancies in the world, is the leading cause of cancer-related deaths in China. Lung cancer is divided into two main types: small cell lung cancer (SCLC) and non-small cell lung cancer (NSCLC). Approximately 80% of lung cancer cases are NSCLC (including adenocarcinoma and squamous cell carcinoma), and the five-year survival rate of NSCLC patients is <15%^1,2^. The invasion and metastasis of malignant tumours are one of the important causes of cancer treatment failure, poor prognosis or death of NSCLC patients. NSCLC metastasis has been recently reported to be caused by a variety of aberrant molecular changes, including the mutational activation of ROS1^3^, MET^4^, HER2^5^, RET, and ALK^6^ oncoproteins and the inactivation of the CDKN2A, RB1, and TP53 tumour suppressor genes^7^. Nevertheless, the comprehensive mechanisms for NSCLC metastasis remain to be further defined.

The suppressor of cytokine signalling 2 (SOCS2) belongs to a family of proteins composed of eight members, SOCS1 to SOCS7 and cytokine-inducible SH2-containing protein CIS. The family of SOCS proteins has been shown to act as negative regulators of cytokine-induced signalling^8^. SOCS proteins appear to switch off JAK/STAT signalling by binding through their SH2 domains to phosphorylated tyrosines within the cytokine receptor and associated JAK complex, or by targeting proteins for proteasomal degradation^8,9^. Several studies illustrate the impact of SOCS2 on certain cancers. For example, SOCS2 could inhibit the activity of Janus kinase 2 (JAK2) and reduce JAK2–STAT3 binding in head and neck squamous cell carcinoma (HNSCC)^10^. Dysregulation of SOCS protein expression can be one of the mechanisms that induce the metastatic potential of hepatocellular carcinoma (HCC) cells in HCC progression^11–13^. However, the biological roles of SOCS2 in NSCLC metastasis have not been investigated.

SOCS2 regulates multiple signalling pathways that are induced by various cytokines such as growth hormone (GH), interleukin-2 (IL-2), and insulin-like growth factor (IGF)^14,15^. The IGF pathway has been extensively studied as an important signalling pathway in cancer. IGF1 and its receptor, type 1 insulin-like growth factor receptor (IGF1R), have been implicated in carcinogenesis, and deregulation of the IGF1R signalling cascade has been described in NSCLC^16^. The yeast two-hybrid data and data from GST-IGF1R pull-down assays demonstrated that SOCS2 can bind to the IGF1R in vitro^15^. The possibility that SOCS2 plays a regulatory role in IGF1R signalling in NSCLC progression warrants investigation.

The current studies, therefore, examined the expression of SOCS2 in NSCLC and corresponding normal tissues. In addition, we investigated the role of SOCS2 in the invasion and metastasis as well as in epithelial–mesenchymal transition (EMT) of lung adenocarcinoma cells in vitro and in vivo. Furthermore, using in vitro assays, we provided evidence that SOCS2 limits the actions of IGF1 or signalling via the IGF1R to reduce EMT, and we explored the possible cellular mechanism. Our results suggest the involvement of SOCS2 in lung adenocarcinoma metastasis and demonstrate its potential use as a biomarker for diagnosing of lung adenocarcinoma.

## Results

### Downregulation of SOCS2 expression correlates with the progression and poor prognosis of NSCLC

**Fig. 1:**
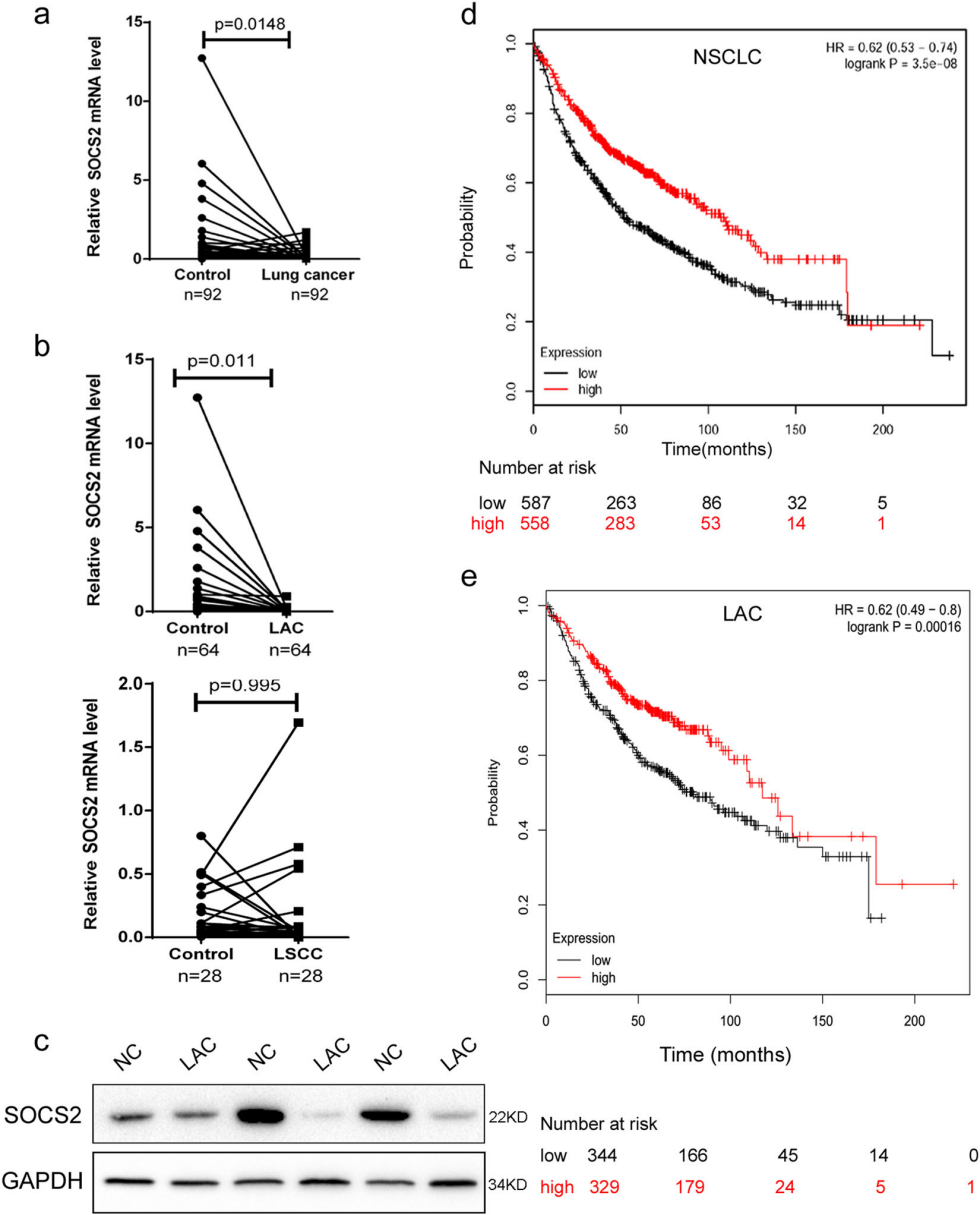
Downregulation of SOCS2 expression correlates with the progression and poor prognosis of NSCLC. **a** Plot diagram showing the SOCS2 mRNA levels in 92 paired human NSCLC samples and paired normal adjacent tissues. **b** Sub-analysis of SOCS2 mRNA levels on lung adenocarcinoma (LAC, *n* = 64) and lung squamous cell carcinoma (LSCC, *n* = 28). **c** Representative example of a western blot of SOCS2 showing samples from paired normal tumour-adjacent lung tissue and lung adenocarcinoma tissue. **d**, **e** Kaplan–Meier curves of NSCLC patients (**d**, *n* = 1882; *p* < 0.0001, log-rank test) and lung adenocarcinoma patients (**e**, *n* = 1104; *p* < 0.001, log-rank test) with high versus low expression of SOCS2

To determine the clinical relevance of SOCS2 expression in patients with NSCLC, the expression levels of SOCS2 were investigated in 92 paired NSCLC samples and paired normal adjacent tissues by real-time PCR and immunoblotting assays. The histopathological results of NSCLC tissue sections are shown in Supplementary Figure S1a. Relative to normal adjacent tissues, SOCS2 expression was significantly downregulated (*P* < 0.01) in 86% (80/92) of tumour tissues (Fig. 1a). Moreover, we observed decrease in SOCS2 levels in tumours compared to normal tissues in 61/64 (95%, *p* = 0.011) of adenocarcinoma and 19/28 (67%, *p* = 0.995) of squamous cell carcinoma (Fig. 1b, c). Moreover, the low expression level of SOCS2 was significantly correlated with the advanced pathological stage (*p* = 0.016) and lymph node metastasis (*p* < 0.001) in NSCLC patients. However, SOCS2 expression was not associated with other parameters such as the tumour size (*p* = 0.4), gender (*p* = 0.835), and age (*p* = 0.277) of NSCLC patients (Table 1). In addition, SOCS2 expression was associated with histological subtype (*p* = 0.002). Further analysis indicated that the expression of SOCS2 negatively correlated with clinical stage (*p* = 0.045) and lymph node metastasis (*p* = 0.013) especially in patients with lung adenocarcinomas (Supplementary Table S1).

**Table 1:** Correlation between SOCS2 expression and clinicopathological characteristics of NSCLC patients (*n* = 92)

Characteristics	SOCS2		*P*
	High no. cases (%)	Low no. cases (%)	Chi-squared test *P*-value
*Age (years)*			0.277
≤65	14 (30.4)	19 (41.3)	
>65	32 (69.5)	27 (58.7)	
*Gender*			0.835
Male	22 (47.8)	23 (50.0)	
Female	24 (52.2)	23 (50.0)	
*TNM stage*			0.016^a^
Ia + Ib	15 (32.6)	7 (15.2)	
IIa + IIb	19 (41.3)	14 (30.4)	
IIIa	12 (26.1)	25 (54.3)	
*Tumour size*			0.400
≤3 cm	28 (60.9)	24 (52.2)	
>3 cm	18 (39.1)	22 (47.2)	
*Lymph node metastasis*			<0.001^a^
Negative	36 (78.2)	15 (32.6)	
Positive	10 (21.8)	31 (67.4)	
*Histological subtype*			0.002^a^
Adenocarcinoma	25 (54.3)	39 (84.7)	
Squamous cell carcinoma	21 (45.7)	7 (15.3)	

^a^ Overall *p* < 0.05

Kaplan–Meier survival analysis and log-rank tests using the overall patient survival were used to further evaluate the relationship between SOCS2 expression and prognosis of lung cancer patients (http://kmplot.com/analysis). According to relative SOCS2 expression in lung cancer tissues, the 1882 patients were classified into two groups: the high SOCS2 group (*n* = 909) and the low SOCS2 group (*n* = 973). The overall survival time of patients with low expression of SOCS2 was significantly shorter than that of patients with high SOCS2 expression (Fig. 1d). Importantly, the Kaplan–Meier Plotter analysis revealed that lung adenocarcinoma patients (*n* = 1104) with low SOCS2 expression levels had worse overall survival (Fig. 1e, *p* < 0.001), whereas the survival curve of patients with lung squamous cell carcinoma (*n* = 444) is unrelated to the expression of SOCS2 (Supplementary Figure S1b). Moreover, the expression of SOCS2 was downregulated in the multiple lung cancer microarray, especially in lung adenocarcinoma by Oncomine database analysis (www.oncomine.org) (Supplementary Figure S1c).

Taken together, these results indicate that downregulation of SOCS2 is correlated with lung adenocarcinoma progression and poor prognosis.

### SOCS2 inhibits migration and invasion of lung adenocarcinoma cells in vitro

**Fig. 2:**
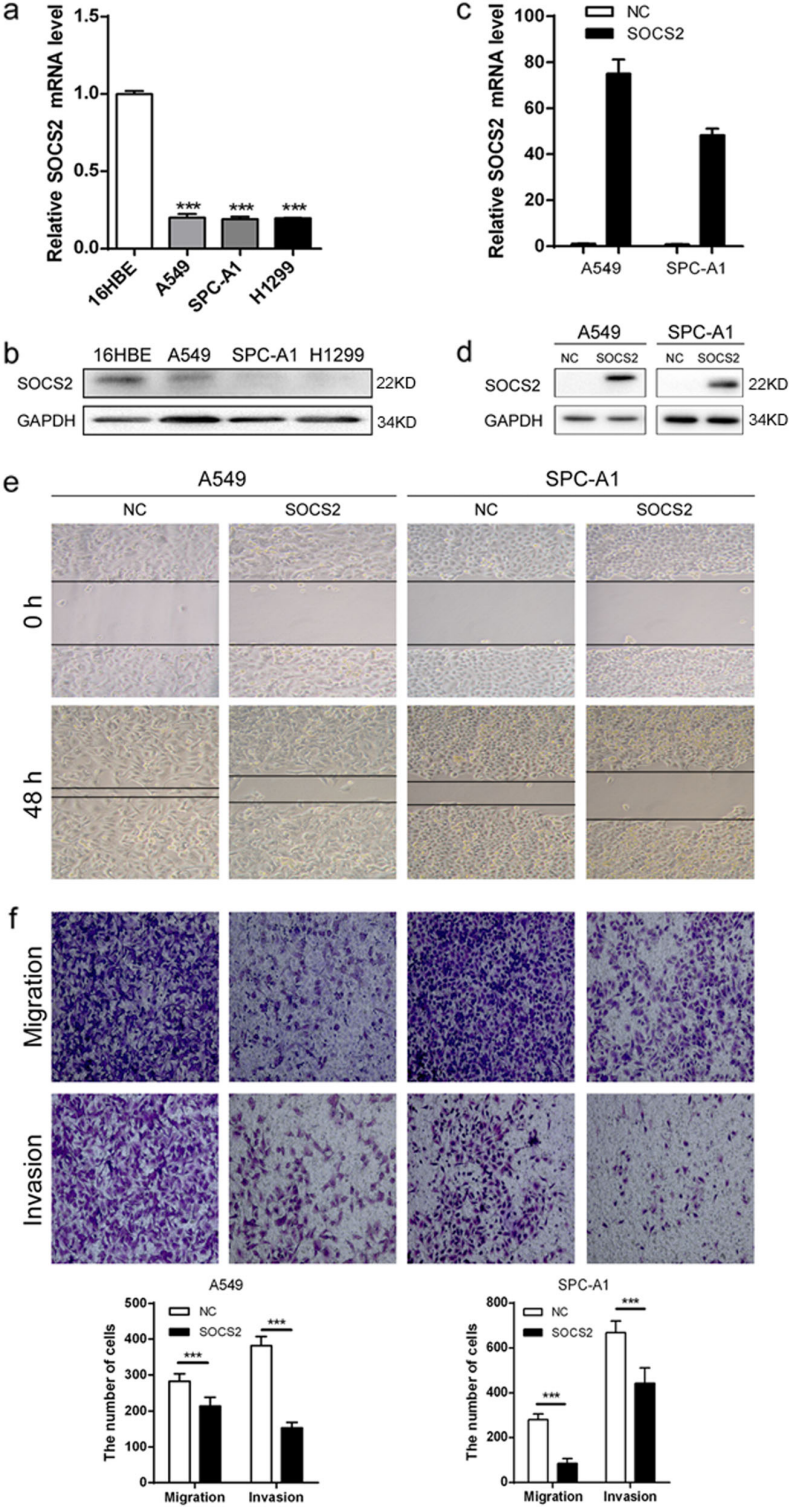
SOCS2 inhibits migration and invasion of lung adenocarcinoma cells in vitro. SOCS2 mRNA (**a**) and protein (**b**) levels were detected in normal human lung cells (16HBE) and lung adenocarcinoma cell lines (A549, SPC-A1, and H1299). Transfection efficacy of SOCS2 overexpression in A549 and SPC-A1 cells was analysed by real-time PCR (**c**) and western blotting (**d**). Error bars indicate the means ± SEM of three experiments. ***p < 0.001. **e** Scratch assays were used to investigate the migratory ability of lung adenocarcinoma cell lines. **f** Evaluating the effects of SOCS2 overexpression on the migration and invasion of A549 and SPC-A1 cells using transwell assays. Error bars represent the means ± SEM (*n* = 5, ***p < 0.001)

We then examined SOCS2 expression in normal lung cells and lung adenocarcinoma cells. As shown in Fig. 2a, b, A549, H1299, or SPC-A1 lung adenocarcinoma cells exhibited lower SOCS2 mRNA and protein levels compared with the levels in normal lung epithelial cell lines (16HBE).

To investigate the role of SOCS2 in lung adenocarcinoma, SOCS2 was overexpressed in A549 or SPC-A1 cells, and the transfection efficiency was confirmed by real-time PCR (Fig. 2c) and immunoblotting (Fig. 2d). Compared with vector pXJ40-HA, both A549-SOCS2 and SPC-A1-SOCS2 cells displayed significant decreases in the migratory and invasive abilities as assessed by the wound healing (Fig. 2e) and transwell assays (Fig. 2f), whereas no significant change was observed in cell proliferation (Supplementary Figure S2a). In addition, there was no significant difference in the results of the colony formation assay (Supplementary Figure S2b), and the upregulation of SOCS2 in A549 and SPC-A1 cells had no effect on cell apoptosis (Supplementary Figure S2c). Furthermore, knockdown of SOCS2 in both A549-SOCS2 and SPC-A1-SOCS2 cells partially restored migratory and invasive efficiencies compared with that of the corresponding control, as revealed by both the wound healing (Supplementary Figure S3a) and transwell assays (Supplementary Figure S3b). The knockdown efficiency of SOCS2 was shown in Supplementary Figure S3c. In particular, both A549-SOCS2 and SPC-A1-SOCS2 cells were significantly more sensitive to the therapy of cisplatin than control cells (Supplementary Figure S4), indicated that lung adenocarcinoma cells with low level of SOCS2 affect chemosensitivity.

### SOCS2 inhibits metastasis of lung adenocarcinoma cells in vivo

**Fig. 3:**
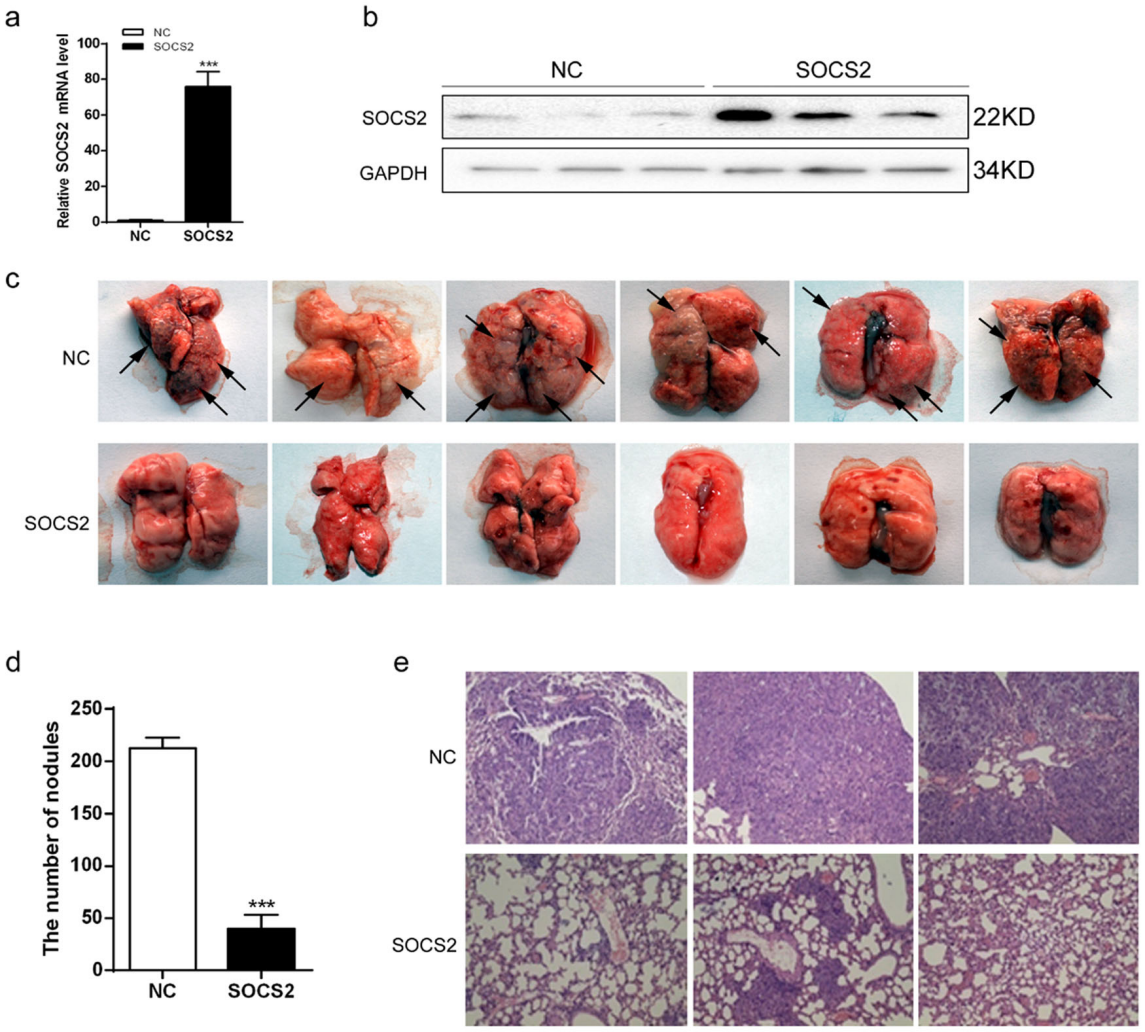
SOCS2 inhibits metastasis of lung adenocarcinoma cells in vivo. **a**, **b** A549 cells overexpressing SOCS2 were transplanted into athymic mice (*n* = 10 per group). The SOCS2 expression in mice lung cells was analysed by real-time PCR (**a**) and western blotting (**b**). Error bars indicate the means ± SEM (*n* = 10, ***p < 0.001). **c**, **d** Number of metastatic nodules on the surface of the lungs of mice injected with control cells or A549-SOCS2 cells was presented. Partial representative nodules were indicated with arrows. The data are represented as the mean nodules numbers ± SEM (*n* = 10, ***p < 0.001). Representative images and H&E (**e**) staining of lungs on day 60 after mice were injected with A549 transfectants (*n* = 10 per group)

To further investigate the effect of SOCS2 on lung adenocarcinoma metastasis in vivo, we injected transfected cell lines, A549-SOCS2 cells, and their corresponding control cells into the lateral veins of nu/nu mice and evaluated their metastatic growth in the lung. After 8 weeks, the efficiency of SOCS2 overexpression in the xenografts was verified by real-time PCR and immunoblot analysis (Fig. 3a, b). As shown in Fig. 3c, d, the A549-SOCS2 cells-injected mice displayed statistically significantly lower numbers of lung metastases (metastatic nodules) than the mice injected with control cells. When lungs underwent haematoxylin and eosin staining, fewer lung metastatic nodes were observed in the mice intravenously injected with A549-SOCS2 cells compared with those observed in the control group (Fig. 3e). Collectively, these results indicate that SOCS2 is necessary for limiting the aggressive and highly metastatic phenotype of lung adenocarcinoma cells.

### SOCS2 regulates the EMT phenotypes in lung adenocarcinoma cells

**Fig. 4:**
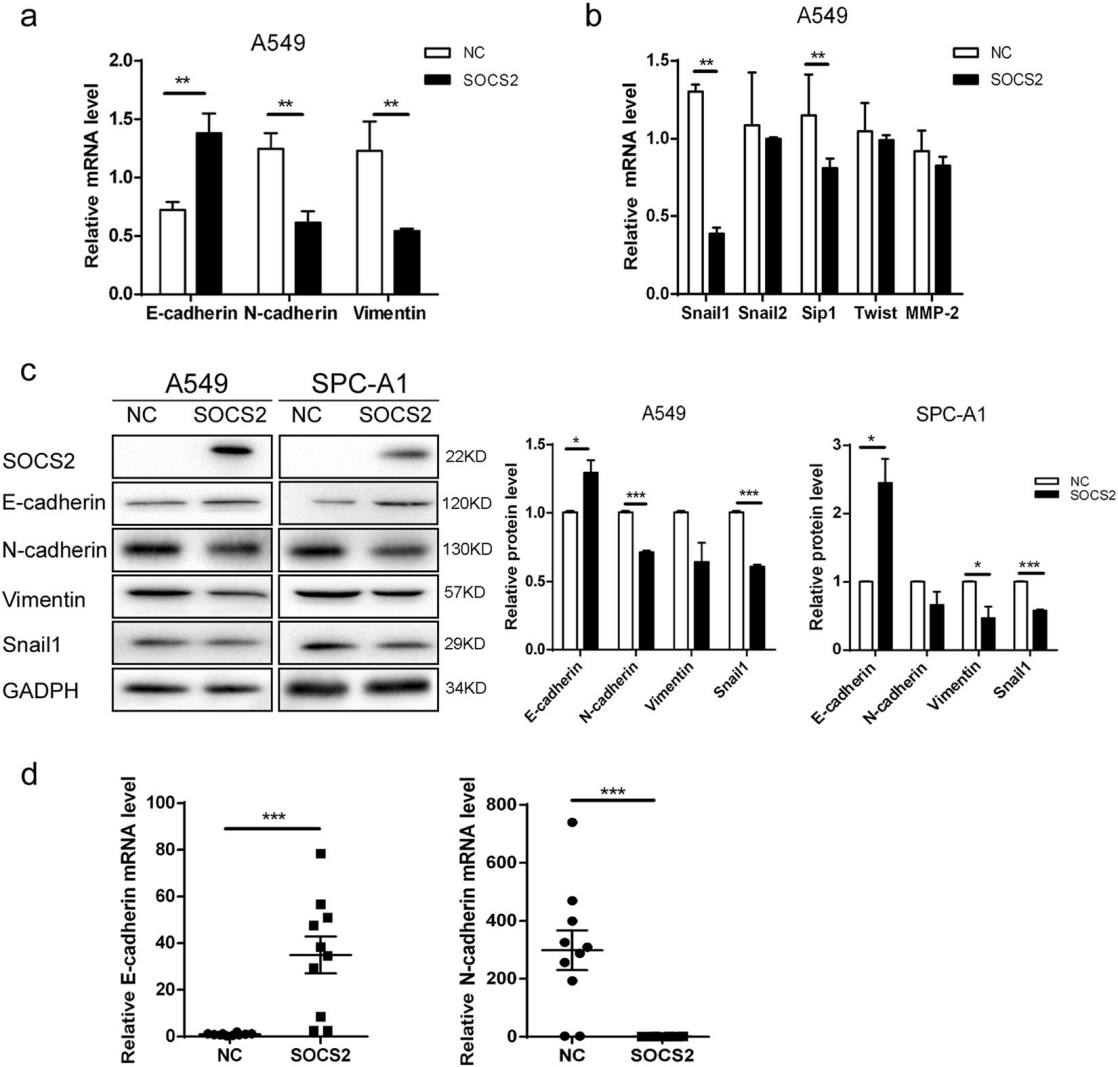
SOCS2 regulates the EMT phenotypes in lung adenocarcinoma cells. **a**, **b** Relative mRNA levels of EMT biomarkers (**a**) and EMT-related transcription factors (**b**) in SOCS2-overexpressed A549 and SPC-A1 cells as indicated. Data are represented as the means ± SEM. **p < 0.01. **c** Representative immunoblots of indicated proteins are shown. The histogram (right panel) represents a densitometric analysis performed to quantify the relative intensity of bands detected by western blotting. Data are represented as the mean ± SEM. *p < 0.05; **p < 0.01; ***p < 0.001. **d** Relative mRNA levels of EMT biomarkers in lungs tissue of mice injected with control cells or A549-SOCS2 cells. The data are represented as the means ± SEM (*n* = 10)

EMT is the initial step of tumour invasion and metastasis. Therefore, we next examined the expression of EMT markers and EMT-related transcription factors in lung adenocarcinoma cells. In A549 cells, SOCS2 overexpression substantially increased the levels of the epithelial marker (E-cadherin) and decreased the levels of the mesenchymal markers (N-cadherin and Vimentin) and EMT-related transcription factors (Snail1 and Sip1), both in mRNA and protein levels (Fig. 4a–c). This observation was further confirmed by the expression of EMT markers and related transcription factors using immunoblot analysis in SPC-A1 cells (Fig. 4c). In contrast, the silenced SOCS2 expression in A549-SOCS2 cells greatly suppressed the expression of E-cadherin and increased the expression of N-cadherin, Vimentin, and Snail1 (Supplementary Figure S5).

The expression of EMT markers was further observed in xenograft tumours from A549-SOCS2 cells (Fig. 4d). Consistent with results from an in vitro study, overexpression of SOCS2 increased the RNA levels of E-cadherin and decreased the levels of N-cadherin and Vimentin.

Therefore, these data together indicate the importance of the SOCS2 in regulating EMT inhibition in lung adenocarcinoma cells.

### SOCS2 limits the actions of IGF1 on the EMT in lung adenocarcinoma cells via the STAT3/STAT5 signalling pathway

**Fig. 5:**
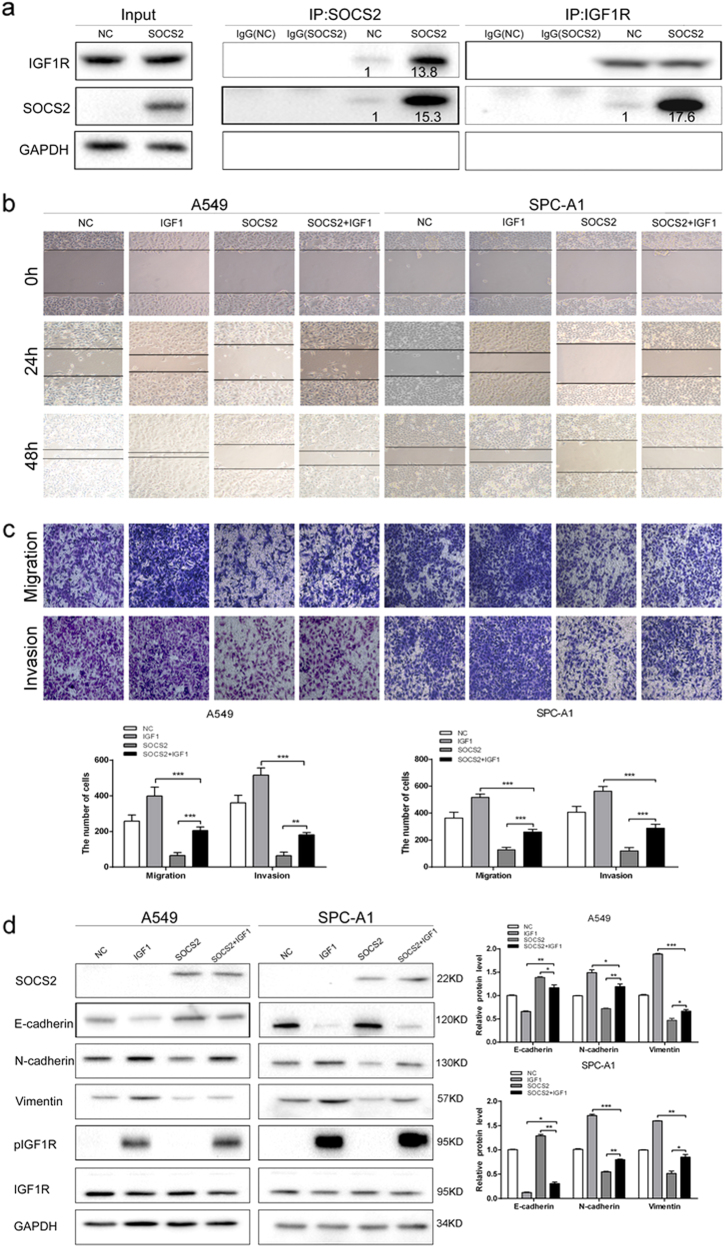
SOCS2 limited the actions of IGF1 on EMT of lung adenocarcinoma cells. **a** Coimmunoprecipitation of SOCS2 with IGF1R using protein lysates from A549-SOCS2 cells. **b**, **c** Evaluating the effects of SOCS2 on the migration and invasion of A549 and SPC-A1 cells in the presence of 200 ng/μL IGF1 using scratch test (**b**) and transwell assays (**c**). Error bars represent the means ± SEM (*n* = 5, **p < 0.01; ***p < 0.001). **d** Immunoblotting analyses of SOCS2, IGF1R and EMT biomarkers in A549 and SPC-A1 cells in the presence of 200 ng/μL IGF1, harbouring SOCS2 plasmid or not. GAPDH was used as a loading control. Representative immunoblots of indicated proteins are shown. The histogram (right panel) represents a densitometric analysis performed to quantify the relative intensity of bands detected by western blotting. Data are represented as the mean ± SEM. *p < 0.05; **p < 0.01; ***p < 0.001

SOCS2 has been shown to interact with the cytoplasmic domain of IGF1R and is thought to be involved in the regulation of IGF1R-mediated cell signalling. To determine whether SOCS2 associates with IGF1R in lung adenocarcinoma cells, immunoprecipitation was performed and as shown in Fig. 5a and Supplementary Figure S6a, an increase in SOCS2 expression corresponded to an increased association between SOCS2 and IGF1R; however, no interaction was observed between SOCS2 and JAK (Supplementary Figure S6b).

Since IGF1 signalling confers the EMT to several types of cancer cells, we further examined whether SOCS2 is involved in the IGF1-induced EMT process of lung adenocarcinoma cells. As shown in Fig. 5b, c, IGF1 treatment effectively promoted the migration and invasion of both A549 and SPC-A1 cells. In contrast, overexpression of SOCS2 in IGF1-treated lung adenocarcinoma cells markedly decreased the migratory and invasive abilities as assessed by the wound healing and transwell assays (Fig. 5b, c). Furthermore, IGF1 treatment could not effectively decrease E-cadherin protein levels and increase the expression of mesenchymal markers in A549-SOCS2 and SPC-A1-SOCS2 cells (Fig. 5d), indicating that SOCS2 is involved in the regulation of IGF1-induced EMT in lung adenocarcinoma cells. However, both the total and phosphorylated IGF1R levels were not changed in A549-SOCS2 and SPC-A1-SOCS2 cells (Fig. 5d).

**Fig. 6:**
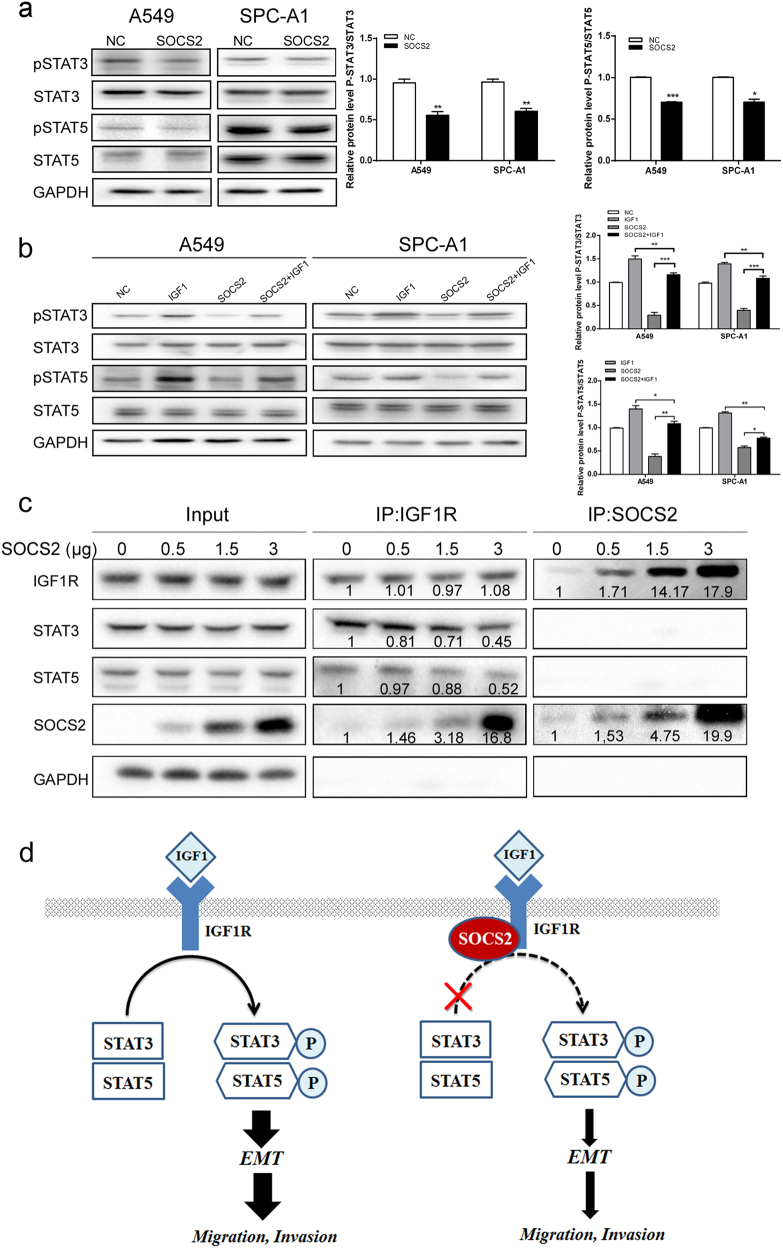
SOCS2 regulates EMT of lung adenocarcinoma cells through STAT3 and STAT5 signalling pathway. **a** The expression of STAT3, pSTAT3, STAT5, and pSTAT5 were analysed in A549-SOCS2 and SPC-A1-SOCS2 cells by western blotting. **b** Immunoblotting analyses of STAT3, pSTAT3, STAT5, and pSTAT5 in A549 and SPC-A1 cells in the presence of 200 ng/μL IGF1, harbouring SOCS2 plasmid or not. Representative immunoblots of indicated proteins are shown. The histogram (right panel) represents a densitometric analysis performed to quantify the relative intensity of bands detected by western blotting. Data are represented as the mean ± SEM. *p < 0.05; **p < 0.01; ***p < 0.001. **c** Coimmunoprecipitation of IGF1R with STAT3 or STAT5 using protein lysates from A549 cells with the increase of SOCS2 expression. GAPDH was used as a loading control. **d** Schematic diagram of the regulatory mechanism of SOCS2 on IGF1R/STAT signalling pathway in lung adenocarcinoma

It has been previously shown in other cell types that STATs can be activated by IGF1R upon IGF1 stimulation. We next explored the role of SOCS2 in IGF1-mediated JAK/STAT signalling. As shown in Fig. 6a, in A549 and SPC-A1 cells, overexpression of SOCS2 strongly reduced both STAT3 and STAT5 phosphorylation, whereas no change in the total protein levels of STAT3 and STAT5 was observed. Depletion of SOCS2 in A549-SOCS2 cells substantially restored the phosphorylation of both STAT3 and STAT5 (Supplementary Figure S7), indicating that both STAT3 and STAT5 activity are primarily subjected to negative regulation by SOCS2 in lung adenocarcinoma cells. Furthermore, SOCS2 was transiently transfected into A549 and SPC-A1 cells along with an empty vector, and STAT3 and STAT5 activation were observed following IGF1 stimulation. As shown in Fig. 6b, both STAT3 and STAT5 tyrosine phosphorylation was inhibited in SOCS2-overexpressed cells but not in IGF1-stimulated cells transfected with empty vector, indicating that SOCS2 is a negative regulator of IGF1-mediated JAK/STAT signalling.ef

To further explain the reduction of IGF1R-mediated STAT3 or STAT5 activation by SOCS2, coimmunoprecipitation was carried out to test the interaction of endogenous IGF1R, STAT3, and STAT5. Treatment of the A549 cells with IGF1 resulted in obvious coimmunoprecipitation of IGF1R with both STAT3 and STAT5. Although no such interaction was detected between SOCS2 and STAT3 or STAT5, a dose-dependent association between IGF1R and STAT3 was found to be reduced with the increase of exogenous SOCS2 expression, suggesting that SOCS2 affected the association pattern between the endogenous STAT3 and IGF1R (Fig. 6c). Similar to the association between of IGF1R and STAT3, the interaction between STAT5 and IGF1R was also reduced with the overexpression of SOCS2 (Fig. 6c). Of note, regardless of the addition of IGF1, SOCS2 could interfere with the combination of STAT3/STAT5 and IGF1R (Supplementary Figure S8).

Taken together, these observations suggest that SOCS2 is involved in the regulation of IGF1R-mediated EMT of lung adenocarcinoma cells, which is probably dependent on the STAT3 and STAT5 pathway.

## Discussion

The SOCS proteins have been identified as potential tumour suppressor proteins^17^. Among the members of the SOCS family, SOCS2 protein has been suggested to have an important role as a feedback inhibitor in certain human cancers; however, its role may be more intricate, as it is variably reported as having both activating and suppressing functions in normal cytokine-induced signalling^18^. As reported, low expression of SOCS2 was associated with hepatocellular, ovarian, breast, and pulmonary cancers^19,20^. In contrast, in bone marrow cells from chronic myeloid leukaemia (CML) patients, SOCS2 is highly expressed and is thus hypothesised to be involved in advanced stages of CML^21^. Based on these controversial findings, we investigated the clinical significance of SOCS2 in NSCLC progression, and its expression negatively correlated with the malignant phenotypes of lung adenocarcinoma. Overexpression SOCS2 reduced lung adenocarcinoma cell migration and invasion in vitro and inhibited metastasis of lung adenocarcinoma cells in vivo by regulating EMT biomarkers, suggesting that reduced SOCS2 promotes the progression of lung adenocarcinoma. Moreover, IGF1 could not effectively induce EMT and tumour malignant development in SOCS2-overexpressed lung adenocarcinoma cells, further indicating the critical inhibitory role of SOCS2 in the IGF1/IGF1R axis in the EMT and progression of lung adenocarcinoma cells. As the EMT is the initial step of tumour metastasis, these data greatly support the concept of SOCS2-involved lung adenocarcinoma metastasis and reveal a novel biological role of SOCS2 in malignant cancer progression. Of note, the silenced SOCS2 expression in A549-SOCS2 cells greatly regulated EMT markers back to control levels of untransfected cells, but still a large effect was observed on migration and invasion, suggested that EMT might be partially involved in the SOCS2 induced changes of invasion, migration and the scratch assay. Prospective follow-up studies are needed to confirm this hypothesis.

The JAK/STAT pathway is critical for cell development, cell survival, cell proliferation, and apoptosis^22,23^. Many cytokines induce the expression of SOCS, which act in a negative feedback loop to prevent further STAT signal transduction^24,25^. Aberrant activation of STAT pathways, particularly of STAT1, STAT3, and STAT5, has been reported in haematological malignancies^26^ and solid tumours, including NSCLC^27–29^. Similarly, our results show that downregulated SOCS2 could induce STAT3 phosphorylation in lung adenocarcinoma cells, whereas exogenous expression of SOCS2 leads to STAT3 inactivation; as such, these results are in agreement with the previous result that reduced expression of SOCS3 causes increased STAT3 phosphorylation in NSCLC lines and tissues^30^. In addition to STAT3, overexpression of SOCS2 protein also inhibits STAT5 phosphorylation in our experiment. Furthermore, SOCS2 overexpression not only reduced the STAT3 and STAT5 transcriptional activity but also reduced the EMT of lung adenocarcinoma cells, suggesting that SOCS2-modulated STAT3 and STAT5 signalling plays an important role in lung adenocarcinoma migration and invasion.

Insulin-like growth factor 1 (IGF1) is a growth and differentiation factor and has been confirmed in various types of cancer cells, including lung cancer cells^31^. Through its receptor (IGF1R), IGF1 promotes the activation of both PI3K/AKT/mTOR and RAS/RAF/MAP kinase^32,33^ which enhances cellular growth^34^. IGF1 binding to IGF1R phosphorylates several targets, such as STAT3^35^ and STAT5^36^, and thus activates the JAK/STAT pathway. A few studies to date have validated components of IGF1 signalling as targets for SOCS2. For example, overexpression of SOCS2 suppresses the actions of IGF1 on rat mesangial cell proliferation, extracellular matrix (ECM) proteins production, and DNA synthesis^37^. Although SOCS2 has been shown to bind to the IGF1R and decrease its biological actions in vitro^15^, the ability of SOCS2 to regulate IGF1 signalling in NSCLC remains to be further defined. In the current study, our data show that SOCS2 is involved in attenuating the IGF1-induced STAT3 and STAT5 activity in lung adenocarcinoma cells. The reduction of SOCS2 leads to continuous enhancement of the activation of IGF1 signalling and its target gene expression, ultimately leading to malignant cellular behaviour. However, differences in invasion are still observed, although pSTAT3/pSTAT5 levels and expression levels of most EMT markers are rescued by SOCS2 overexpression in IGF1-treated cells, indicated that SOCS2 might partially regulated IGF1R signalling.

It has been suggested that SOCS might play a role in negative regulation of receptor tyrosine kinase signalling by insulin and IGF1 receptors. SOCS2 has been shown to play an important role in the IGF1 signalling pathway in the brain; however, the precise mechanism is not clear^15^. SOCS proteins inhibit the JAK/STAT pathways by binding, through their SH2 domains, to the phosphorylated tyrosine residues within the cytokine receptor or within the associated JAK complex. SOCS proteins can also promote proteasomal degradation of the JAKs. In our experiments, the function of SOCS2 was distinct from its classically understood role in haematopoietic cells. SOCS2 inhibited STAT activity, but there was no interaction between SOCS2 and JAK in A549 cells, which was possibly due to the loss of the classic kinase inhibitory region that SOCS1 and SOCS3 proteins possess^38^. SOCS2 is also classically understood to promote the degradation of JAK2; however, we did not observe changes in total STAT3 and STAT5 or JAK2 levels in A549 cells following SOCS2 overexpression. Instead, SOCS2 was associated with IGF1R, which is consistent with previously reported yeast two-hybrid results^15^. Most interestingly, the expression of SOCS2 dose dependently reduced the interaction between IGF1R and STAT3 or STAT5, indicating that SOCS2 acts as a STAT3/STAT5 inhibitor that can competitively bind to IGF1R and thus can attenuate the IGF1-induced STAT3/STAT5 activity in lung adenocarcinoma cells. It has been reported that SOCS2 appears to be involved in the competitive binding to the STAT5 and SHP2 binding sites on the GH receptor, which further confirmed the different inhibitory mechanism of SOCS2 in our experiment.

As a novel molecular target, IGF1R is gradually known for the treatment of NSCLC^39,40^. Several studies have demonstrated IGF1R plays a key role in the survival of cancer cell and causes resistance to a variety of anticancer drugs^41^. However, to date, the drugs for targeting SOCS2 proteins still have not been discovered. Considering that the low expression of SOCS2 in NSCLC, HNSCC, hepatocellular carcinoma, and oral squamous cell carcinoma with distant metastasis and that the overwhelming majority of NSCLC patients succumb to their disease as a result of distant metastasis, our study suggests a promising therapeutic target for lung adenocarcinoma in clinical practice and probably in the other malignant tumours. We could try to screen for candidate inducers for targeting SOCS2 in a panel of FDA-approved drugs using homology modelling.

In conclusion, this study presents the pivotal finding that low expression of SOCS2 induces invasion and metastasis of human lung adenocarcinoma cells by regulating EMT both in vitro and in vivo, which is mainly dependent on the IGF1/IGF1R-stimulated STAT3 or STAT5 pathway. These findings provide insights into the underlying molecular mechanism of the malignant progression of SOCS2-mediated lung adenocarcinoma.

## Materials and methods

### Patient data and tissue samples

All patients, their relatives or both provided written informed consent for their clinical and pathological information to be used for research, and this information was stored in the hospital database. The Ethics Committee of Nanjing Medical University approved the methods and experimental protocols used in the present study, which were performed in accordance with the ethical standards of our institutional research committee and the tenants of the 1964 Declaration of Helsinki and its later amendments or comparable ethical standards. Methods were performed in accordance with the approved guidelines. With informed consent, a total of 92 primary NSCLC tissues and paired normal adjacent tissues were selected from patients who underwent resection of NSCLC at the First Affiliated Hospital of Nanjing Medical University (Nanjing, China) between 2016 and 2017. The patients were diagnosed with NSCLC by histopathological examination, and none of these patients was treated with local or systemic therapy before surgery. All tissue samples were stored in liquid nitrogen.

### Cell culture

Three lung adenocarcinoma cell lines (A549, SPC-A1, NCI-H1299) and a normal human bronchial epithelial cell line (16HBE) were purchased from the Institute of Biochemistry and Cell Biology of the Chinese Academy of Sciences (Shanghai, China). The A549, NCI-H1299, and 16HBE cell lines were cultured in RPMI 1640; the SPC-A1 cell line was cultured in DMEM (GIBCO-BRL, MD, USA). These media were supplemented with 10% foetal bovine serum (FBS), 100 U/mL penicillin, and 100 mg/mL streptomycin (Invitrogen, Carlsbad, CA). All cells were cultured in humidified air with 5% CO_2_ at 37 °C. For the exogenous IGF1 induction experiment, A549 and SPC-A1 were serum-starved overnight and then treated with fresh RPMI 1640 or DMEM containing 0.5% FBS and 200 ng/mL IGF1 (Sigma Aldrich, MO, USA) for 24 h before proceeding to downstream experiments.

### Plasmids, transfection, and RNA interference

The SOCS2 (GenBank accession number NM003877) coding sequence was amplified by PCR with indicated primers (Supplementary Table S2) and inserted into the pXJ40-HA vector. Then, the plasmid expressing SOCS2 was transfected into the indicated cells using Lipofectamine 2000 (Invitrogen, Carlsbad, CA). An empty pXJ40-HA vector was used as a control. SOCS2-specific small interfering RNA (siRNA) (Supplementary Table S2) and scrambled siRNA were synthesised from Invitrogen according to a previous report and were transfected into cells using Lipofectamine 2000 reagent.

### Cell viability assays

The protocols and reagents (Sigma Aldrich, MO, USA) used for the MTT assay (detection of cell proliferation/viability) were all strictly conducted according to previous work^42^.

### Real-time PCR and western blotting

RNA isolation, cDNA synthesis, quantitative PCR with indicated primers (Supplementary Table S2), and western blotting were performed as previously described^42^. The antibodies used in this study include the following: E-cadherin, 610182; N-cadherin, 610920 (BD Biosciences, CA, USA); SOCS2, 2779; Vimentin, 9775; STAT5, 94205; pSTAT5, 4322; STAT3, 4904; pSTAT3, 9145; IGF1R, 9750; pIGF1R, 3021; JAK2, 3230; Snail1, 3879 and GAPDH, 2118 (Cell Signaling Technology, MA, USA).

### Scratch test

For the scratch test, cells from each group were inoculated into six-well plates at 5.0 × 10^4^ cells per well. When cell confluence reached 90%, the cells were starved overnight in serum-free medium. Three parallel lines were scratched on the bottom of the culture plate using a sterile 20 μL micropipette tip. The cells were washed twice using serum-free medium and cultured for another 48 h. The changes in cell motility were observed and were imaged under an Olympus inverted fluorescence phase-contrast microscope (×40). Experiments were carried out in triplicate, and three randomly selected fields of each well were recorded.

### Cell migration and invasion assays

For the transwell assays, cells (5 × 10^4^ cells for migration; 1 × 10^5^ cells for invasion) were transferred into the upper chamber of an insert (8-μm pore size; Millipore, Billerica, MA, USA) at 48 h post transfection. Medium with 10% FBS was added to the lower chamber. Cells were cultured for 24 h, and then, cotton wool was used to remove the cells that remained on the upper membrane. Next, cells were stained with methanol and 0.1% crystal violet. Cell imaging and counting were conducted with a Leica DM IRB Inverted Research Microscope (LEICA, Germany). All experiments were performed in triplicate, and results were expressed as the average number of cells per field (*n* = 5).

### Xenograft studies

Male athymic mice (5 weeks old) were purchased from the Animal Center of the Nanjing University (Nanjing, China) and were maintained in laminar flow cabinets under specific pathogen-free conditions. The tumour metastatic ability of A549-SOCS2 cells and control cells (2 × 10^7^ cells per cell line) was observed following intravenous injection of cells into the tail vein (*n* = 10 per group). After 8 weeks, the mice were sacrificed and the number of metastatic nodules on the lung surface was counted. Metastatic lung samples were fixed with 4% paraformaldehyde before dehydration and paraffin embedding. Paraffin sections were stained with haematoxylin and eosin according to standard protocols. This study was carried out in strict accordance with the Guide for the Care and Use of Laboratory Animals of the National Institutes of Health. The protocol was approved by the Committee on the Ethics of Animal Experiments of Nanjing Medical University (Permit Number: 1601238). All surgeries were performed under sodium pentobarbital anaesthesia, and all efforts were made to minimise suffering.

### Co-immunoprecipitation

Co-immunoprecipitation assay was performed as previously described^42^.

### Statistical analysis

Statistical analysis was conducted using Student’s *t*-test for simple comparisons between two groups and one-way ANOVA for comparisons between multiple groups using SPSS 17.0 (IBM, IL, USA) and GraphPad Prism Ver. 6.01 (San Diego, CA, USA). The relationship between SOCS2 expression and clinical characteristics was analysed using the *χ*^2^ test. Survival curves were plotted by the Kaplan–Meier method and compared using the log-rank test. All data are expressed as the mean ± SEM. Differences were considered significant at *p* < 0.05 (*p < 0.05; **p < 0.01; ***p < 0.001).

### Ethics statement

All patients and/or their relatives provided written informed consent for their clinical and pathological information to be used for research and to be stored in the hospital database; this study, including its methods and experimental protocols, was approved by the Ethical Committee of the First Affiliated Hospital of Nanjing Medical University (Nanjing, China). All procedures performed in our study were done so in accordance with the ethical standards of our institutional research committee and the 1964 Helsinki Declaration and its later amendments or comparable ethical standards. Methods were carried out in accordance with the approved guidelines.

## Electronic supplementary material

supplementary data(DOCX 3690 kb)
