# Supplementary material for: Suppressor of cytokine signalling-2 limits IGF1R-mediated regulation of epithelial–mesenchymal transition in lung adenocarcinoma
Source: Cell Death Dis. 2018 Mar 20;9(4):429. doi: 10.1038/s41419-018-0457-5 (PMC5861121; doi:10.1038/s41419-018-0457-5)

**Supplementary Table 1. Correlation between SOCS2 expression and clinicopathological characteristics of** **lung adenocarcinoma patients (*n*=64). * Overall *p*<0.05.**

| **Characteristics** | **SOCS2** |  | ***P*** |
| --- | --- | --- | --- |
|  | High No. cases (%) | Low No. cases (%) | Chi-squared test *P*-value |
| **Age(years)** |  |  | 0.802 |
| ≤65 | 17 (51.5) | 15 (48.3) |  |
| >65 | 16 (48.5) | 16 (51.7) |  |
| **Gender** |  |  | 0.135 |
| Male | 13 (39.3) | 18 (58.1) |  |
| Female | 20 (60.7) | 13 (41.9) |  |
| **TNM Stage** |  |  | 0.045* |
| Ia + Ib | 9 (27.3) | 4 (12.9) |  |
| IIa + IIb | 13 (39.4) | 8 (25.8) |  |
| IIIa | 11 (33.3) | 19 (61.3) |  |
| **Tumor size** |  |  | 0.161 |
| ≤3cm | 21 (63.6) | 14 (45.1) |  |
| >3cm | 12 (36.4) | 17 (54.9) |  |
| **Lymph node metastasis** |  |  | 0.013* |
| Negative | 22 (66.7) | 11 (35.4) |  |
| Positive | 11 (33.3) | 20 (64.6) |  |
|  |  |  |  |

**Supplementary Table 2. Primers for qPCR and sequences of siRNA used in this study.**

| Strand/Gene | Primer | Sequence(5’-3’) |
| --- | --- | --- |
| *SOCS2* | SOCS2F | TTAAAAGAGGCACCAGAAGGAAC |
|  | SOCS2R | AGTCGATCAGATGAACCACACT |
| *E-cadherin* | E-cadherinF | CGAGAGCTACACGTTCACGG |
|  | E-cadherinR | GGGTGTCGAGGGAAAAATAGG |
| *N-cadherin* | N-cadherinF | CCCTGCTTCAGGCGTCTGTA |
|  | N-cadherinR | TGCTTGCATAATGCGATTTCACC |
| *Vimentin* | VimentinF | AGTCCACTGAGTACCGGAGAC |
|  | VimentinR | CATTTCACGCATCTGGCGTTC |
| *Snail1* | Snail1F | TCGGAAGCCTAACTACAGCGA |
|  | Snail1R | AGATGAGCATTGGCAGCGAG |
| *Snail2* | Snail2F | TGTGACAAGGAATATGTGAGCC |
|  | Snail2R | TGAGCCCTCAGATTTGACCTG |
| *Sip* | SipF | GCGATGGTCATGCAGTCAG |
|  | SipR | CAGGTGGCAGGTCATTTTCTT |
| *Twist* | TwistF | GTCCGCAGTCTTACGAGGAG |
|  | TwistR | GCTTGAGGGTCTGAATCTTGCT |
| *MMP-2* | MMP2F | CGTCTGTCCCAGGATGACATC |
|  | MMP2R | TGTCAGGAGAGGCCCCATAG |
| *Actin* | ActinF | GAAATCGTGCGTGACATTAA |
|  | ActinR | AAGGAAGGCTGGAAGAGTG |
| Oligoname | Sequence (sense strand) | |
| siSOCS2-1 | Sense GCACCAGAAGGAACUUUCUUGAUUA | |
|  | Antisense UAAUCAAGAAAGUUCCUUCUGGUGC | |
| siSOCS2-2 | Sense AACUAAUCU UCGAAUCGAATT | |
|  | Antisense UUCGAUUCGAAGAUUAGUUGG | |

**Supplementary Figure 1. The expression of SOCS2 was downregulated in lung cancer.** (a) H&E staining of human lung adenocarcinoma samples and paired normal adjacent tissues. (b) Kaplan-Meier curves of lung squamous cell carcinoma patients (*n*=271; *p*=0.93, log-rank test) with high versus low expression of SOCS2. (c) The expression of SOCS2 was downregulated in multiple lung cancer microarray by Oncomine database analysis ([www.oncomine.org](http://www.oncomine.org)). Median Rank: it applies to meta-analysis. When comparing results from multiple individual datasets, the results are ranked based on the median *p*-value rank of a gene across the selected analyses.


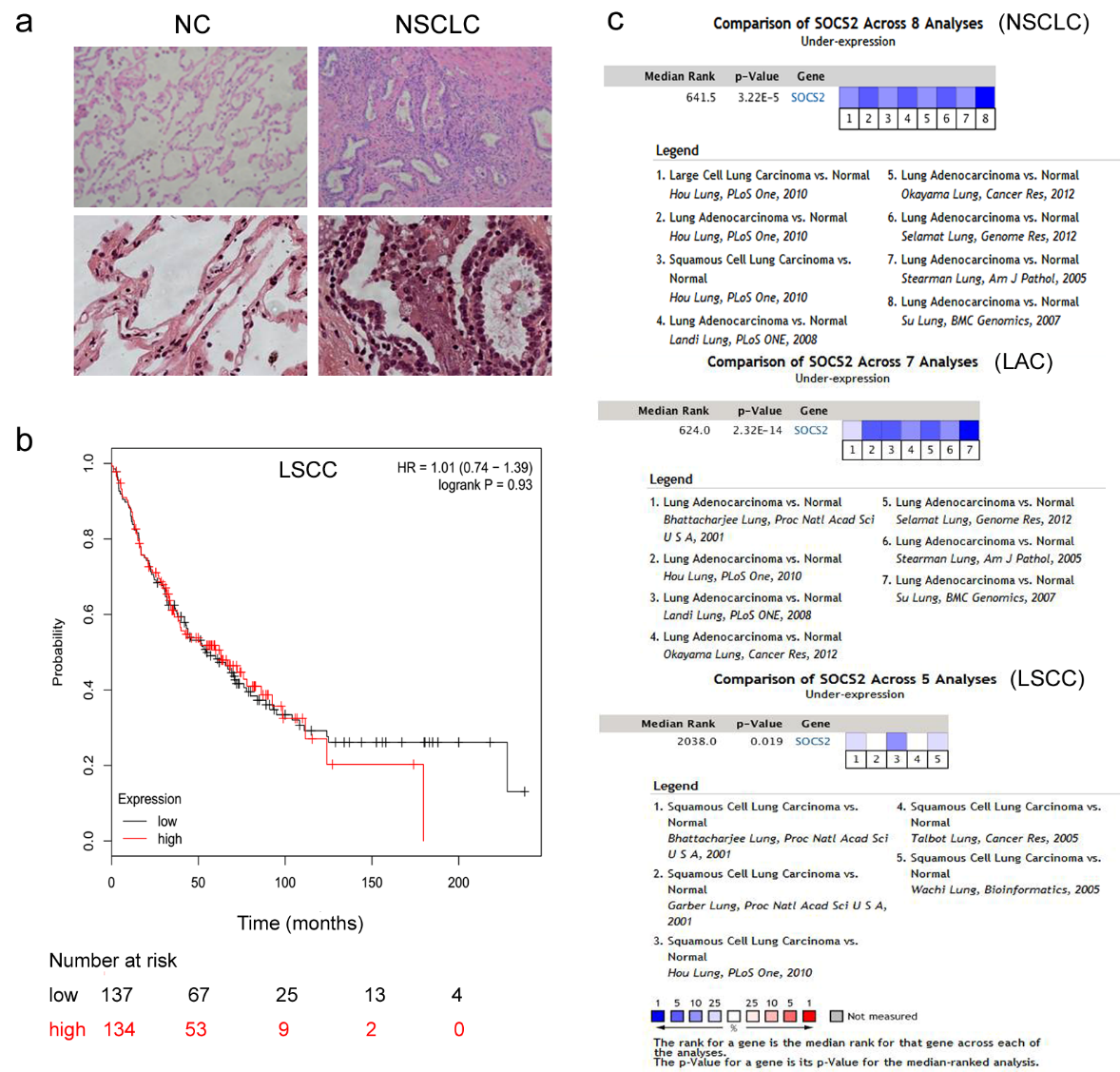


**Supplementary Figure 2. The effect of SOCS2 on** **proliferation and apoptosis of lung adenocarcinoma cells *in vitro*.** (a) Evaluating the effects of SOCS2 overexpression on the growth of A549 and SPC-A1 cells using MTT assay. Error bars, mean ± SEM of three independent experiments. (b) Evaluating the effects of SOCS2 overexpression on the growth of A549 and SPC-A1 cells using Colony‑forming growth assay. The colonies were counted and captured. The data are represented as the means ± SEM of three samples. (c) Evaluating the effects of SOCS2 overexpression on apoptosis of A549 and SPC-A1 cells using flow cytometry.


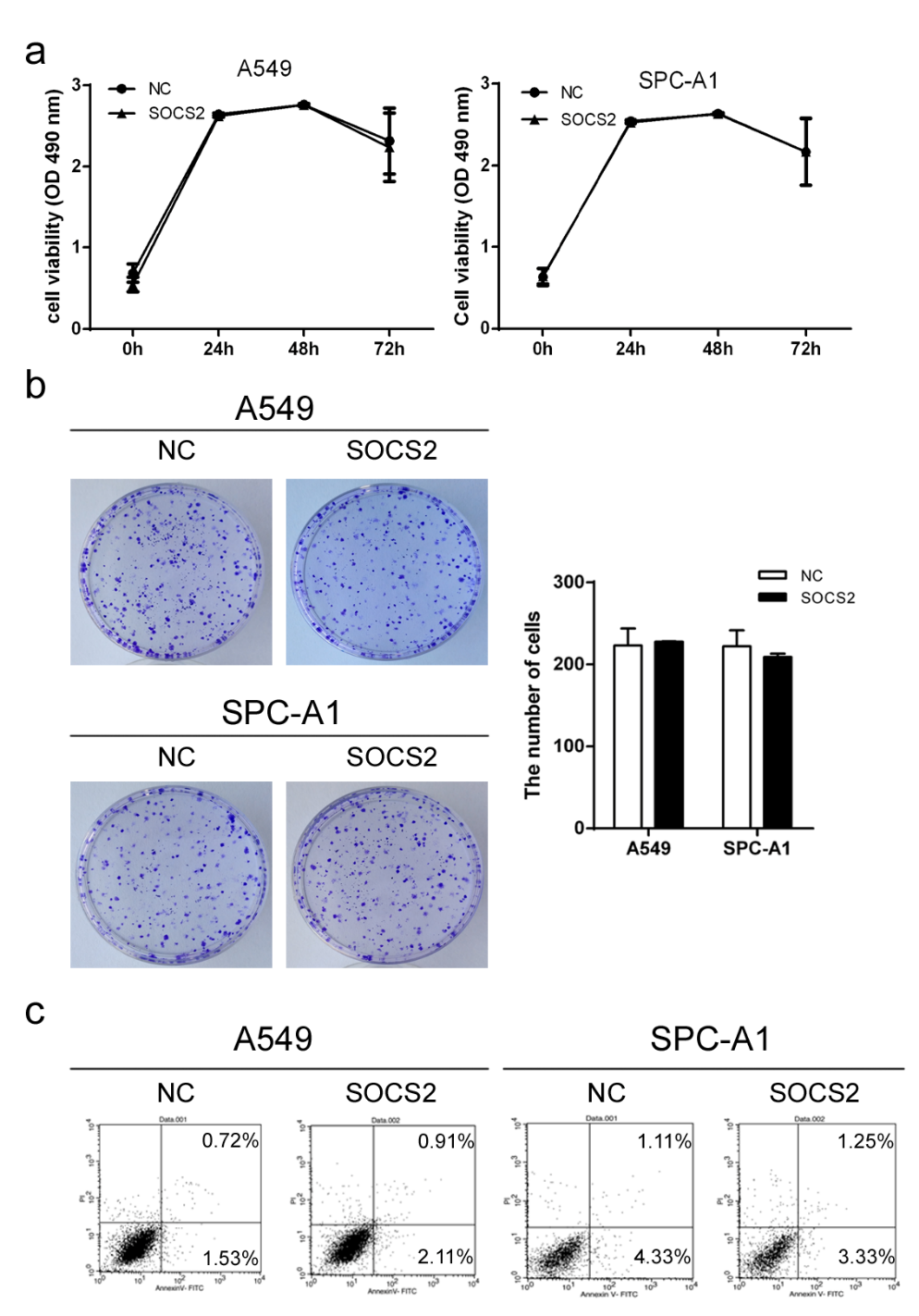


**Supplementary Figure 3. Effects of SOCS2 on chemosensitivity of lung adenocarcinoma cells *in vitro*.** The MTT assay showed that the A549 and SPC-A1 cells transfected with SOCS2 were significantly more sensitive to the therapy of cisplatin than control cells (**p* < 0.05). Data are mean ± SEM of three experiments.


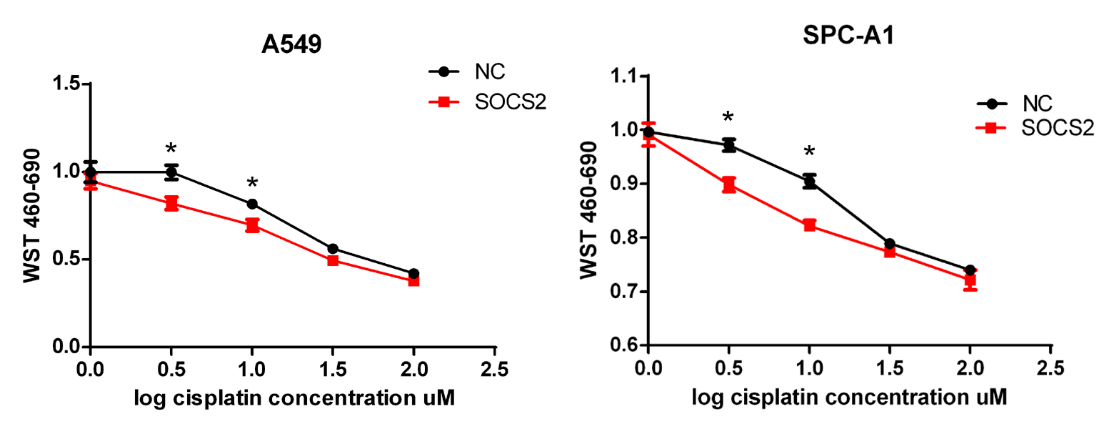


**Supplementary Figure 4. Effects of SOCS2 on migration and invasion of NSCLC cells *in vitro*.** Evaluating the effects of SOCS2 siRNA on the migration and invasion of control, A549-SOCS2 and SPC-A1-SOCS2 cells using Wound-healing assays (a) and Transwell assays (b). Error bars represent the means ± SEM (*n*=5, ****p*<0.001). (c) Transfection efficacy of SOCS2 siRNA in NSCLC cell lines (A549 and SPC-A1) was analyzed by qRT-PCR. siSOCS2 represent the mix siRNA of siSOCS2-1 and siSOCS2-2. Error bars, mean ± SEM of three independent experiments.


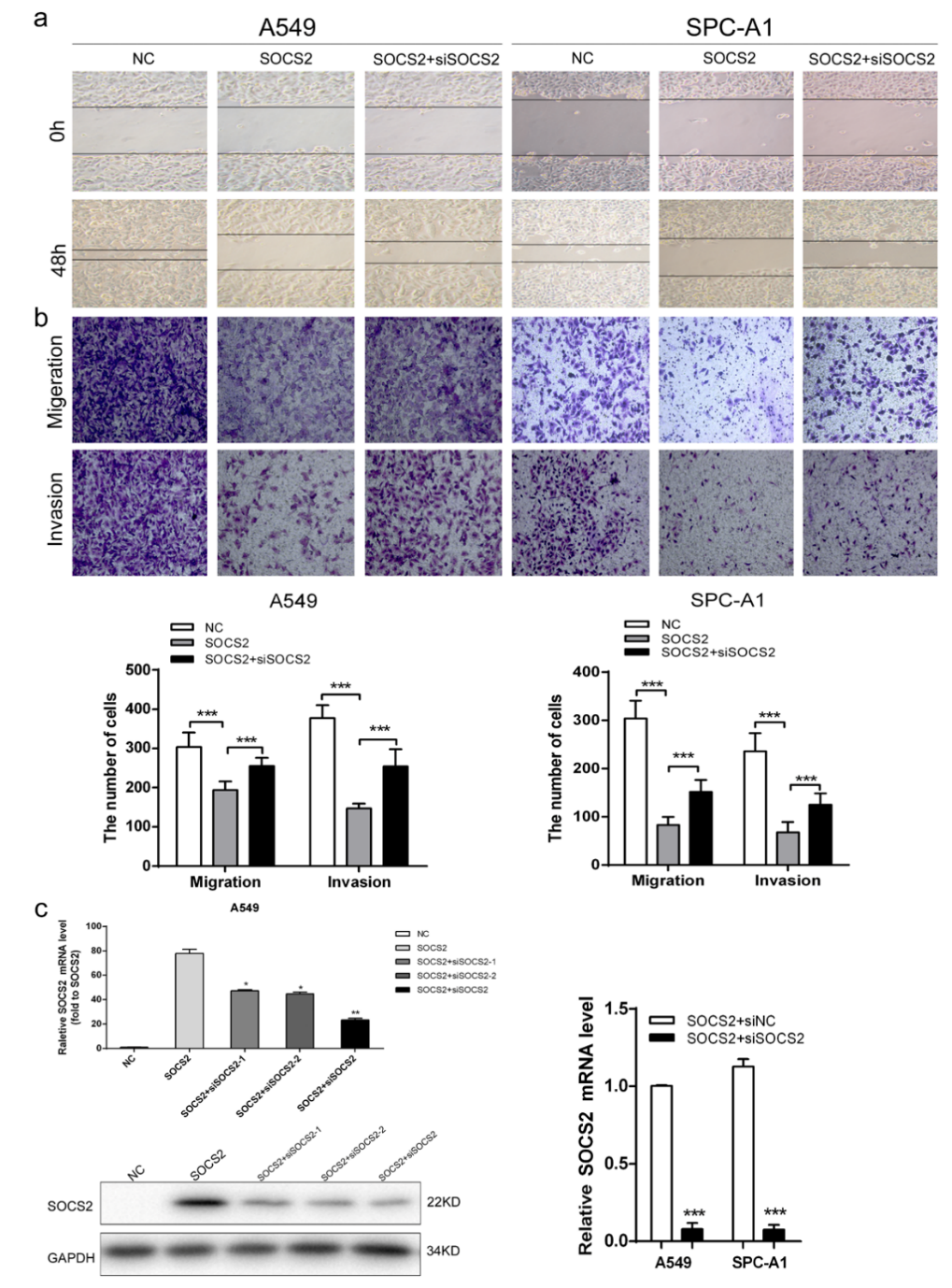


**Supplementary Figure 5. Effects of the SOCS2 on EMT in A549 cells.** Immunoblotting analyses of EMT biomarkers expression in A549-SOCS2 cells harboring SOCS2 siRNA. GAPDH was used as a loading control. The histogram (right panel) represents a densitometric analysis performed to quantify the relative intensity of bands detected by western blotting. Data are represented as the mean ± SEM. *, *p* < 0.05.


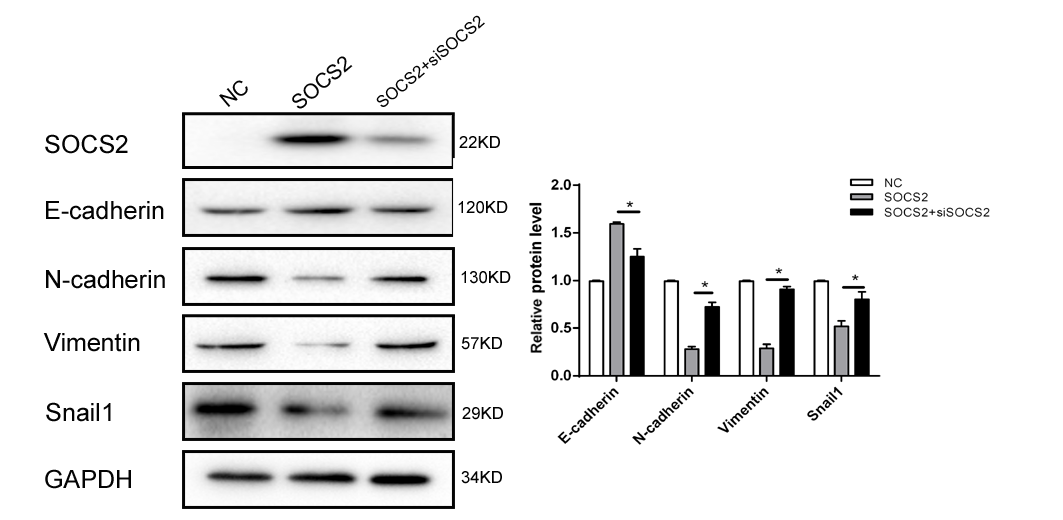


**Supplementary Figure 6. Interaction between SOCS2 and JAK2 in A549 cells.** (a) Coimmunoprecipitation of HA-tag antibody with IGF1R or SOCS2 using protein lysates from A549-SOCS2 cells. (b) Coimmunoprecipitation of JAK2 with IGF1R or SOCS2 using protein lysates from A549-SOCS2 cells. GAPDH was used as a loading control.


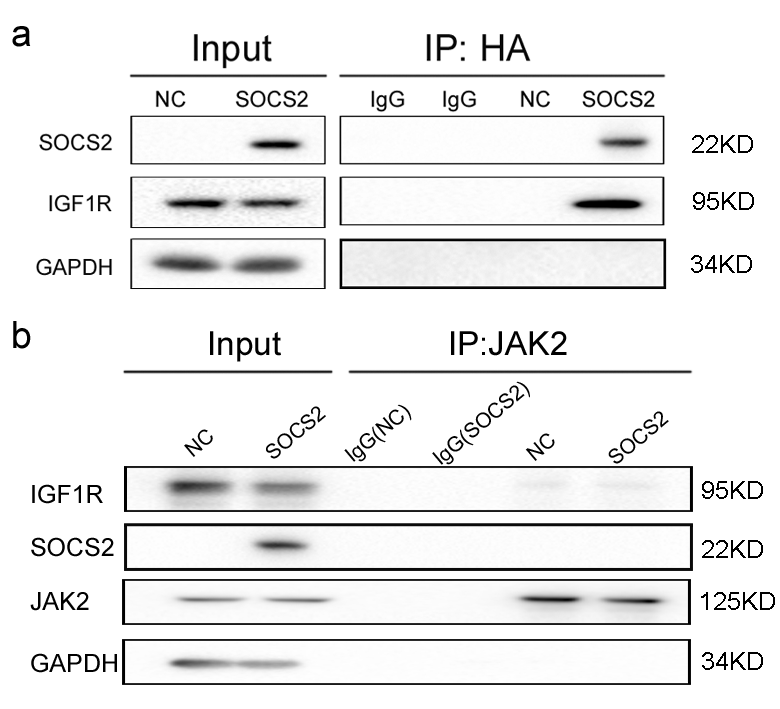


**Supplementary Figure 7. SOCS2 expression negatively regulates STAT3 and STAT5 activation.** Immunoblotting analyses of pSTAT3, STAT3, pSTAT5 and STAT5 expression in A549-SOCS2 cells harboring SOCS2 siRNA. GAPDH was used as a loading control. The histogram (right panel) represents a densitometric analysis performed to quantify the relative intensity of bands detected by western blotting. Data are represented as the mean ± SEM. *, *p* < 0.05.


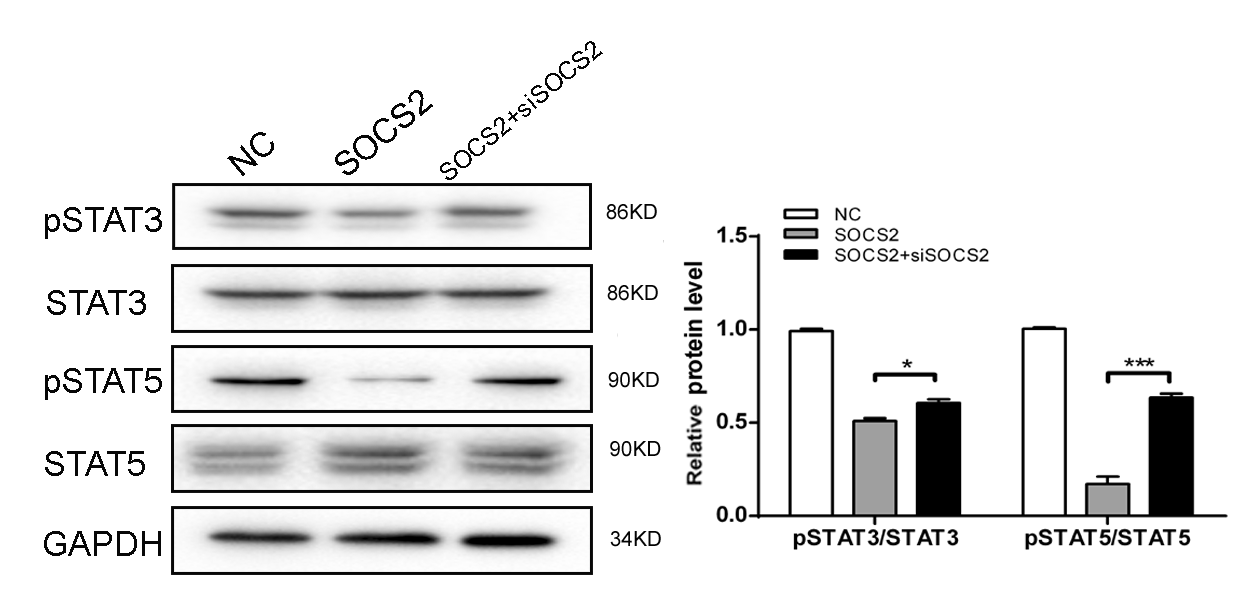


**Supplementary Figure 8. Interaction between IGF1R and STAT3/STAT5 in A549 cells.** Coimmunoprecipitation of IGF1R with STAT3 or STAT5 using protein lysates from both A549 and A549-SOCS2 cells in the presence of 200 ng/μL IGF1 or not. GAPDH was used as a loading control.


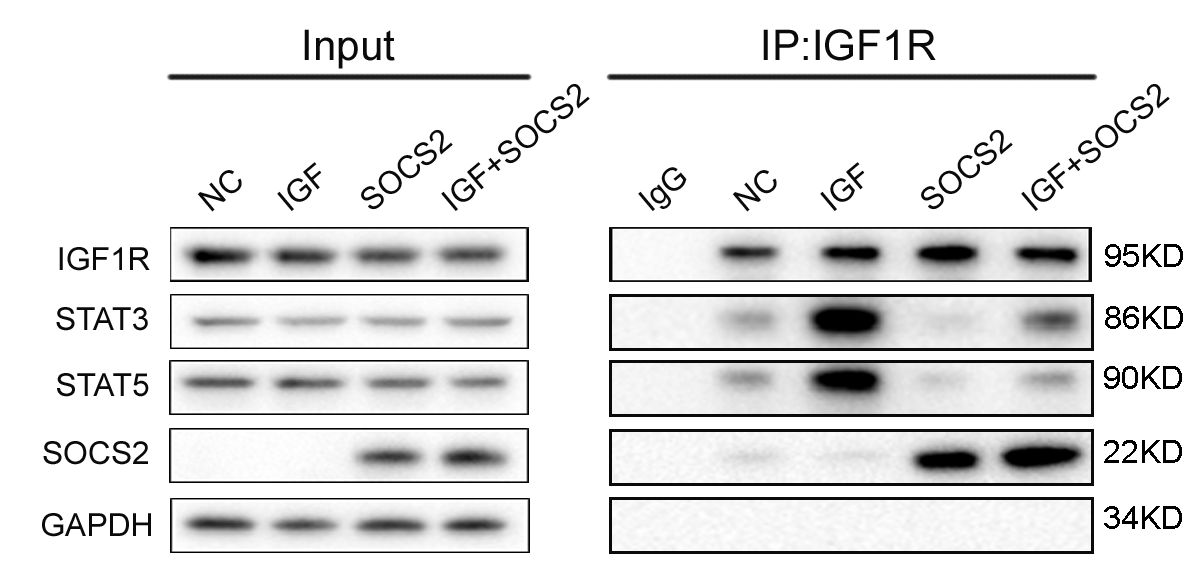

Supplement: Supplementary file 1 — supplementary data(DOCX 3690 kb) [file 41419_2018_457_MOESM1_ESM.docx]
